# Exceptional Chitin Preservation Driven by Early‐Stage Silicification in Mesozoic Freshwater Environments

**DOI:** 10.1111/gbi.70067

**Published:** 2026-10-08

**Authors:** Taro Yoshimura, Kentaro Nakayama, Kaito Asato, Yoshikazu Noda, Takenori Sasaki

**Affiliations:** ^1^ The University Museum, the University of Tokyo Tokyo Japan; ^2^ Faculty of Science and Technology Keio University Yokohama City Kanagawa Prefecture Japan; ^3^ Fukui Prefectural Dinosaur Museum Katsuyama City Fukui Prefecture Japan

**Keywords:** biopolymer degradation, chitin preservation, freshwater bivalves, molecular palaeontology, Taphonomy

## Abstract

Chitin is a fundamental structural biopolymer, yet its fossil record is predominantly biased towards marine Lagerstätten or specific stagnant non‐marine settings, leaving its stability in dynamic continental environments poorly understood. Here, we report the molecular preservation of indigenous chitin within the shells of the bivalve *Nakamuranaia soni* from the Lower Cretaceous Kitadani Formation, Japan—a renowned dinosaur‐bearing fluvial succession. While Paleozoic and Jurassic records of chitin exist for arthropods and brackish gastropods, our findings represent one of the earliest direct records of chitinous biopolymers within a strictly freshwater fluvial Lophotrochozoan. High‐resolution FE‐SEM and histochemical staining (Calcofluor White and Congo Red) revealed exceptionally preserved periostracal honeycomb‐like templates, morphologically and chemically congruent with extant unionids. FT‐IR spectroscopy confirmed diagnostic polysaccharide backbone linkages and amide modes matching reference *β*‐chitin, while host‐rock analysis (negative control) established the indigenous nature of these organics. Decalcification experiments yielded a rigid silica–chitin hybrid framework, suggesting that early‐stage silicification acted as a ‘taphonomic shield’. We propose that rapid mineralisation, driven by volcaniclastic‐rich hydrochemistry, encapsulated the microfibrils, providing both steric hindrance, and chemical stabilisation. This discovery demonstrates that authigenic silicification can mitigate the degradative effects of freshwater diagenesis, significantly expanding the spatial and molecular boundaries of the terrestrial fossil record.

## 1 Introduction

The preservation of organic biopolymers in the fossil record provides critical insights into the evolutionary history and physiological traits of extinct organisms (Cody et al. 2011; Briggs and Summons 2014). Among these macromolecules, chitin—a fibrous homopolymer of β‐(1,4)‐linked N‐acetyl‐D‐glucosamine—is of paramount significance. As the second most abundant natural polysaccharide after cellulose, chitin serves as a critical structural component in arthropod exoskeletons, molluscan shell matrices, and fungal cell walls (Focher et al. 1990; Ehrlich, Janussen, et al. 2008; Ehrlich, Koutsoukos, et al. 2008). Despite its inherent chemical resilience, chitin is typically deemed highly susceptible to microbial degradation, particularly via chitinolytic enzymes, and rapid hydrolysis under conventional diagenetic conditions (Gooday 1990; Flannery et al. 2001). Consequently, the long‐term geochemical survival of chitinous architectures across geological timescales has historically been regarded as an exceptional, if not anomalous, occurrence.

Over the past two decades, sophisticated analytical advancements have facilitated the identification of chitinous remnants in various Palaeozoic and Mesozoic marine Lagerstätten, such as the Cambrian sponge (Ehrlich et al. 2013) and Jurassic gastropod fossils (Wysokowski et al. 2014), as well as Palaeozoic non‐marine arthropods (Astrop et al. 2015). However, such reports remain predominantly confined to marine or stagnant lacustrine settings. Dynamic freshwater fluvial ecosystems, by contrast, are often characterised by higher microbial turnover, fluctuating pH levels, and distinct hydrochemical regimes that are ostensibly more deleterious to the preservation of organic nitrogenous compounds (Meyers 1994; Gupta et al. 2006). This taphonomic bias has resulted in a significant paucity of data regarding the molecular preservation of freshwater invertebrates (Briggs and Summons 2014; Yoshimura, Hirota, et al. 2026), leaving the temporal limits of chitin longevity in continental fluvial Lophotrochozoans largely unexplored.

The Kitadani Formation (Tetori Group) in Fukui, Japan, represents a celebrated Lower Cretaceous (Aptian–Albian) succession (Maeda 1958), renowned for its diverse vertebrate assemblage, including dinosaurs (Shibata and Azuma 2015) and mammals (Miyata et al. 2016). This formation, primarily comprising fluvial and lacustrine sediments, yields an abundance of well‐preserved freshwater molluscs (Asato et al. 2022; Yoshimura, Nakayama, et al. 2026; Yoshimura, Nakayama, Asato, et al. 2026). Whilst the general palaeontology and sedimentology of the Kitadani Formation have been extensively documented (Sano 2015), the molecular composition of its invertebrate remains has, until now, remained unexamined.

Here, we report the preservation of organic biopolymers within the freshwater bivalve *Nakamuranaia soni* (Yang, 1975) (Unionida: Nakamuranaiadidae) from the Lower Cretaceous Kitadani Formation (Figure 1). The shell architecture of *N. soni* exhibits a microstructural sequence typical of unionid bivalves, consisting of an outer organic periostracum, an intermediate prismatic primary layer, and an inner nacreous secondary layer (Figure 1D). This occurrence represents one of the earliest direct records of chitinous material within a strictly freshwater fluvial Lophotrochozoan, expanding the documented environmental context of molecular preservation. Utilising dual‐fluorescence histochemical staining and Fourier‐transform infrared spectroscopy (FT‐IR), we demonstrate the structural and chemical retention of the original molecular architecture. We identify a micrometre‐scale siliceous film coating the honeycomb‐like periostracal templates—a feature providing definitive morphological and chemical evidence of its molluscan origin. We propose that this early‐stage silicification acted as a robust physical and chemical barrier—a taphonomic shield (Butterfield 2003)—which inhibited microbial penetration and stabilised the biopolymers against degradative pore waters. This discovery not only extends the documented temporal range of freshwater chitin preservation but also elucidates the critical role of authigenic mineralisation in preserving labile organic molecules within dynamic continental basins.

**FIGURE 1:**
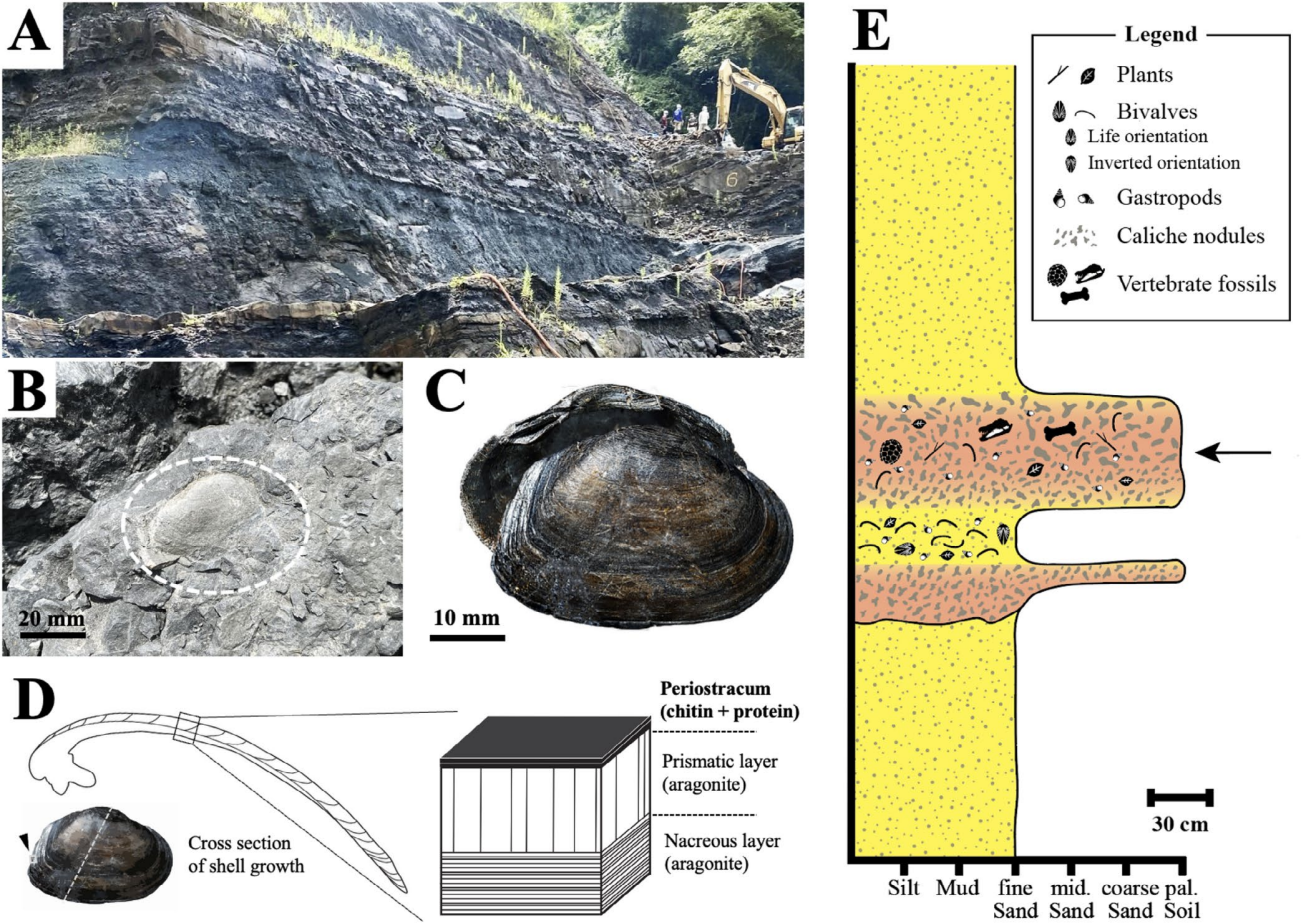
Field occurrence, macromorphology, and shell skeletal architecture of *Nakamuranaia soni* from the Lower Cretaceous Kitadani Formation. (A) Field photograph of the active excavation at the Kitadani dinosaur quarry (Tetori Group) in Katsuyama City, Fukui Prefecture, central Japan. The outcrop exposes a rhythmically bedded succession of alternating sandstones and organic‐rich grey siltstones, reflecting fluctuating fluvio‐lacustrine depositional conditions. (B) In situ occurrence of *N. soni* (enclosed by the white dashed ellipse) within the organic‐rich siltstone matrix. (C) Macroscopic view of an exceptionally preserved specimen of *N. soni*. (D) Schematic diagram illustrating the orientation of the shell growth cross‐section (dashed line and arrowhead) and a three‐dimensional block model detailing the pristine molluscan skeletal microstructures, comprising the outer organic periostracum (chitin–protein matrix), the intermediate prismatic layer (aragonite), and the inner nacreous layer (aragonite). (E) Detailed lithostratigraphic column of the dinosaur‐bearing fluvial–lacustrine sequence of the Kitadani Formation (Aptian–Albian, ca. 120 Ma). The horizontal black arrow indicates the specific high‐energy bonebed horizon yielding the examined fossil unionid assemblages associated with diverse terrestrial vertebrate fauna, charophytes, and carbonised plant remains.

## 2 Materials and Methods

### 2.1 Sample Material and Preparation

Fossil specimens of the freshwater bivalve *Nakamuranaia soni* (Yang, 1975) (Unionida: Nakamuranaiadidae) were collected from the Lower Cretaceous Kitadani Formation (Tetori Group) in Katsuyama City, Fukui Prefecture, Japan (Figure 1). For comparative molecular and microstructural analyses, extant specimens of the unionid bivalve *Nodularia douglasiae* (Gray, 1833) were obtained from Lake Biwa, Shiga Prefecture, Japan. The soft tissues of the extant samples were removed, and the shells were rinsed with deionised water and air‐dried at room temperature prior to analysis. For standard comparison, native *β*‐chitin was isolated from the gladius of the squid *Uroteuthis edulis* (Hoyle, 1885) following mild deproteinisation in 1 M NaOH (room temperature, 24 h) and subsequent neutralisation with deionised water. All examined specimens are registered as voucher specimens and housed in the collections of The University Museum, The University of Tokyo (UMUT).

### 2.2 Optical and Digital Microscopy

To document the macroscopic to micro‐scale surface textures, colour, and mineralogical morphology of the authigenic silica crust on unpolished fossil surfaces, high‐resolution digital optical microscopy was performed using a Leica digital microscope Leica DMS1000 (Leica Microsystems, Wetzlar, Germany). Observations were carried out under calibrated coaxial and ring‐light LED illumination with focus‐stacking to achieve high‐depth‐of‐field imaging of the translucent microcrystalline silica aggregates.

### 2.3 Scanning Electron Microscopy (FE‐SEM and EDS)

The fossilised shells and matrices were examined using a ZEISS MERLIN compact field‐emission scanning electron microscope (FE‐SEM). To mitigate charging effects on non‐conductive fossil surfaces while preserving organic signatures, observations were conducted in low‐vacuum mode (N_2_, 40 Pa) at an accelerating voltage of 15 kV and a working distance of 7 mm. A Variable Pressure Secondary Electron (VPSE) detector was employed to obtain high‐resolution topographic images at magnifications ranging from 200× to 2500×.

Elemental characterisation and spatial mapping were performed using an Oxford Instruments X‐Max energy‐dispersive X‐ray detector integrated with the FE‐SEM system. For each specimen, area mappings and high‐resolution line scans were acquired across the shell cross‐sections to determine the distribution of silicon (Si). Line scans were conducted across the lamellar structures at a resolution of 512 points, with a dwell time of 1000 ms per point to ensure sufficient signal‐to‐noise ratios for trace organic detection.

Quantitative elemental analyses were processed using the Oxford Instruments Aztec software, applying the XPP matrix correction (an evolution of the ZAF method) to account for absorption and fluorescence effects within the heterogeneous siliceous‐organic matrix. For elemental mapping, data were collected at a resolution of 512 × 384 pixels to correlate the concentration of Si with the preserved chitinous lamellae identified in the secondary electron images.

### 2.4 Fourier‐Transform Infrared Spectroscopy (FT‐IR)

FT‐IR spectroscopy was performed on the fossilised shell samples to identify the diagnostic functional groups of chitin and associated proteins. Infrared absorption spectra were acquired using a Bruker INVENIO R spectrometer. For sample preparation, powdered fossil material, finely ground in an agate mortar, was mixed with spectroscopic‐grade potassium bromide (mini KBr plate, Jasco Engineering Co. Ltd.) to form a translucent pellet. A pure KBr pellet was used for background measurements. Spectra were collected in transmission mode with a resolution of 4, averaging 100 scans over the range of 600 to 4000 cm^−1^. Data processing and baseline corrections were carried out using OPUS 8.2.28 software. Spectral analyses focused on identifying both the diagnostic peptide modes (amide I and II bands) and the polysaccharide backbone vibrations, including the *β*‐(1,4)‐glycosidic linkage (896–898 cm^−1^) and asymmetric C–O–C bridge stretching (1157 cm^−1^), to determine the structural integrity and polymorphic form (*β*‐chitin) of the preserved organic fossil.

### 2.5 Histochemical Staining and Fluorescence Microscopy

To confirm the presence and distribution of chitinous biopolymers, histochemical staining was employed using Calcofluor White (CFW) and Congo Red (CR). For CFW staining, sections were first equilibrated in a 0.1 M Tris–HCl buffer (pH 8.5) for 30 min to optimise binding efficiency. The samples were subsequently incubated in a 0.1% (w/v) CFW solution (Fluorescent Brightener M2R, Sigma‐Aldrich) for 45 min in darkness. CFW is a non‐specific fluorochrome that binds to β‐linked polysaccharides, including chitin, emitting a diagnostic blue fluorescence.

Parallel sections were stained with Congo Red (1.5 mg/mL) for 24 h at room temperature, following the procedure established by Michels and Büntzow (2010). CR exhibits a high affinity for chitinous microfibrils and provides a complementary visual signal to CFW. Following incubation, all samples were thoroughly rinsed with distilled water to remove residual solute and dried under ambient conditions.

Fluorescence imaging was performed using a Keyence BZ‐9000 epifluorescence microscope. CFW‐stained samples and unstained control sample were observed using a UV filter set (excitation: 365 nm; emission: 447 nm) with a fixed exposure time of 1/500 s to evaluate signal specificity against background autofluorescence. CR‐stained samples were examined using a green/red filter set (excitation: 540 nm; emission: 605 nm). Identical illumination and acquisition parameters were maintained across stained and unstained specimens to ensure direct comparability and preclude non‐specific autofluorescence artifacts. For high‐resolution topographic and fluorescence correlation, a laser confocal microscope (Olympus LEXT OLS4000) was utilised, enabling the precise spatial localisation of organic remnants within the mineralised framework.

## 3 Results

### 3.1 Microstructural Preservation of the Periostracum

Detailed FE‐SEM observations revealed the exceptional preservation of the periostracum, the outermost organic layer of the shell (Figure 2). In *Nakamuranaia soni*, this layer remains structurally intact at the microscale. Notably, the inner surface of the preserved periostracum exhibits a distinct honeycomb‐like morphology, consisting of polygonal chambers approximately 20–50 μm in diameter (Figure 2E). These structures represent the organic templates that originally enveloped the aragonitic prisms of the outer shell layer. The dimensions and geometric configuration of this honeycomb framework are morphologically congruent with the periostracal architecture observed in the extant unionid *Nodularia douglasiae* (Figure 2C,D). High‐magnification FE‐SEM inspection further resolves the detailed ultrastructure of the fossilised chamber walls and boundary junctions, demonstrating uncollapsed, intact organic wall margins that mirror the pristine morphology of modern specimens (Figure 2D,F).

**FIGURE 2:**
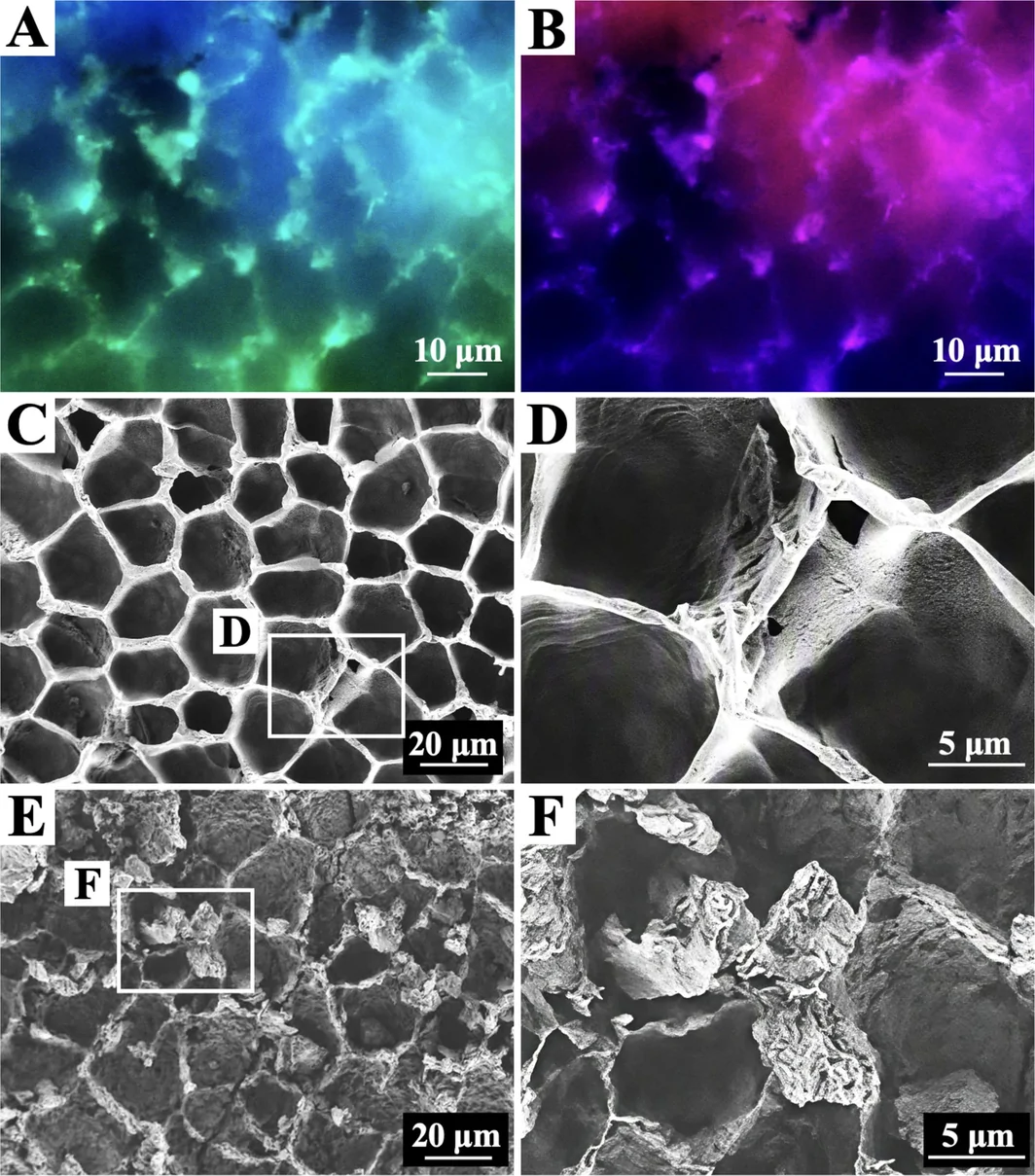
Histochemical and ultrastructural validation of preserved chitinous matrices in the periostracum of *Nakamuranaia soni*. (A, B) High‐magnification epifluorescence micrographs (scale bar = 10 μm) of the fossilised periostracum of *Nakamuranaia soni* demonstrating the presence of indigenous biopolymers through dual‐fluorescence histochemical assays. (A) Calcofluor White (CFW) staining showing intense blue/cyan fluorescence under ultraviolet excitation, diagnostic of β‐linked polysaccharides. (B) Congo Red (CR) staining exhibiting vibrant red fluorescence under green excitation, confirming high affinity for chitinous microfibrils. (C–F) Field‐emission scanning electron microscopy (FE‐SEM) micrographs detailing the microstructural topography and high‐magnification ultrastructure of inner periostracal surfaces. (C, D) The pristine honeycomb‐like organic template in the extant freshwater unionid *Nodularia douglasiae* at low magnification and high magnification, illustrating the polygonal chamber walls that originally enveloped aragonitic outer shell prisms. (E, F) Exceptionally preserved honeycomb‐like frameworks in the Cretaceous *Nakamuranaia soni* at low magnification and high magnification, displaying high ultrastructural fidelity and uncollapsed chamber wall boundaries congruent with modern counterparts.

### 3.2 Molecular and Histochemical Validation

The indigenous nature of the chitin was further supported by comparative FT‐IR and fluorescence analyses. FT‐IR spectra of the fossil periostracum revealed vibrational bands congruent with both extant *N. douglasiae* and the reference *β*‐chitin standard (Figure 3A–C). Specifically, the spectrum resolved a distinct single Amide I band at 1650 cm^−1^ (characteristic of the parallel‐chain *β*‐polymorph, distinct from the split doublet of α‐chitin) and an Amide II band at 1550 cm^−1^. Furthermore, characteristic absorption bands corresponding to the polysaccharide backbone were preserved, including the asymmetric *β*‐(1,4) C–O–C bridge stretching at 1157 cm^−1^ and the diagnostic *β*‐(1,4)‐glycosidic bond vibration at 896–898 cm^−1^ (Figure 3A). Although saccharide ring modes (1030–1070 cm^−1^) were partially masked by authigenic silica overlap in the fossil material, the overall spectral profile matches that of the *β*‐chitin standard. In contrast, the host rock (negative control) yielded only flat baselines devoid of organic absorption bands across the entire 800–1800 cm^−1^ range (Figure 3D).

**FIGURE 3:**
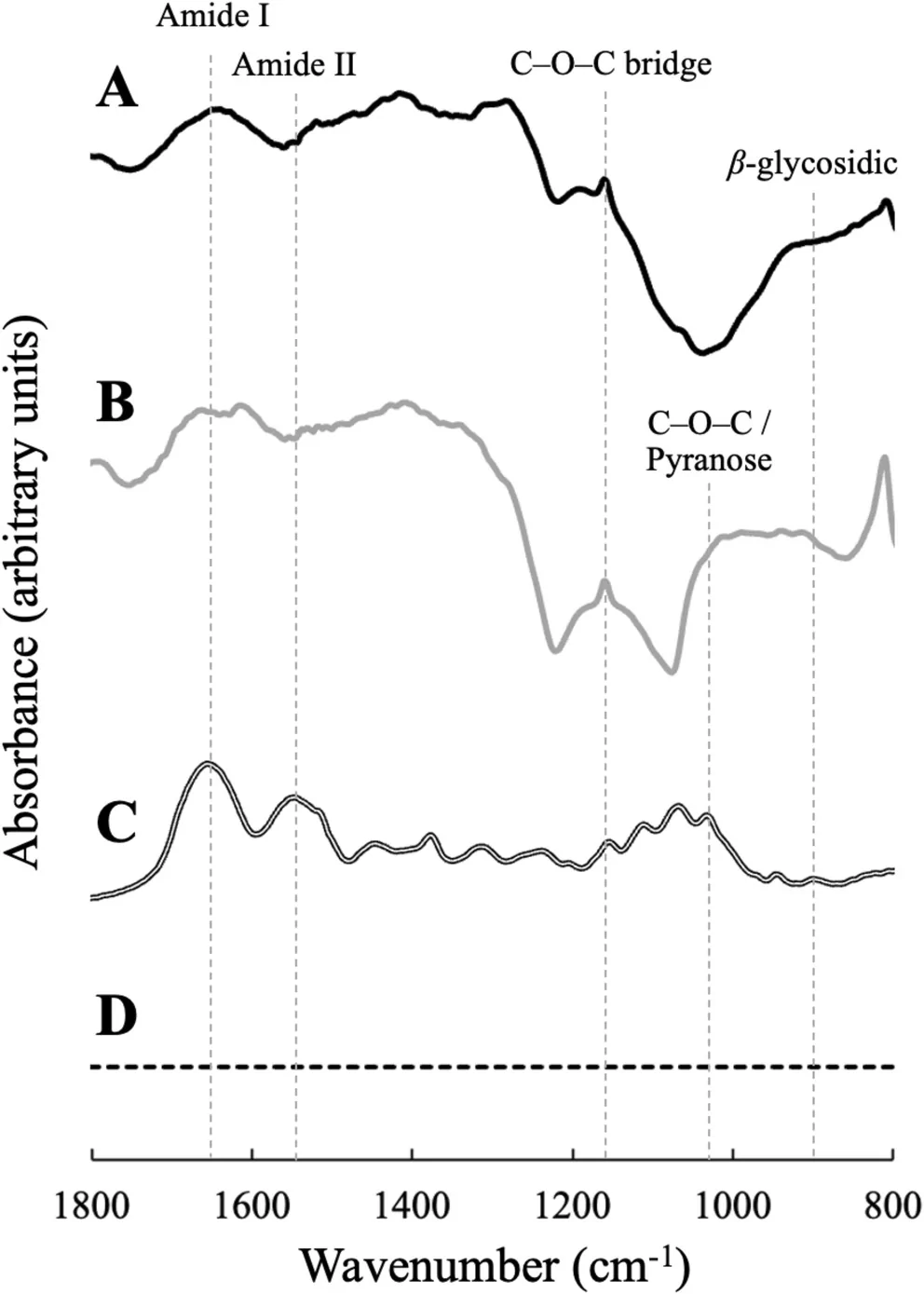
FT‐IR spectra (1800–800 cm^−1^) of fossil and extant periostracal matrices compared with reference *β*‐chitin and sedimentary control. (A) Fossil periostracum of *Nakamuranaia soni*. (B) Extant unionid periostracum of *Nodularia douglasiae* . (C) Reference *β*‐chitin standard isolated from squid gladius. (D) Surrounding sedimentary rock matrix (negative control). Vertical dashed lines indicate key vibrational modes: Amide I (1650 cm^−1^; diagnostic single band characteristic of *β*‐chitin), Amide II (1550 cm^−1^), the *β*‐(1,4) C–O–C bridge stretching (1157 cm^−1^), saccharide pyranose ring vibrations (1030–1070 cm^−1^; partially obscured in fossil periostracum due to authigenic silica overlap), and the diagnostic *β*‐(1,4)‐glycosidic bond (896–898 cm^−1^). Spectra are vertically offset for clarity.

Similarly, high‐magnification histochemical staining provided spatial confirmation of this distinction (Figure 2A,B). Under UV and green/red excitation, the shell's periostracum exhibited intense blue (CFW) and red (CR) fluorescence, respectively (Figure 2A,B). Specifically, the honeycomb‐like templates within the periostracum showed the strongest fluorescent response, highlighting the high concentration of preserved chitinous microfibrils in these structural zones. Under identical acquisition and short exposure parameters (1/500 s), unstained control sections displayed only faint, negligible background autofluorescence (Figure S1), confirming that the intense fluorescence was specific to dye‐fluorochrome binding rather than non‐specific proteinaceous autofluorescence. Furthermore, no corresponding fluorescence was observed in the mineral grains of the surrounding matrix, precluding the possibility of non‐specific binding or background interference from the sedimentary environment.

### 3.3 Characterisation of the Siliceous Coating and Shell–Matrix Interface

Elemental characterisation via EDS mapping on the external surface of the fossil shell demonstrated the prominent development of a continuous, well‐defined siliceous coating. At the anteroventral margin, elemental mapping of silicon (Si) revealed a dense, extensive concentration of the mineral phase tightly overlapping the outer shell architecture (Figure 4A). High‐magnification optical microscopy of this targeted micro‐region confirmed that this authigenic silica phase exhibits a translucent, microcrystalline aggregate character, which physically encapsulates and seals the outermost organic‐rich layers (Figure 4B).

**FIGURE 4:**
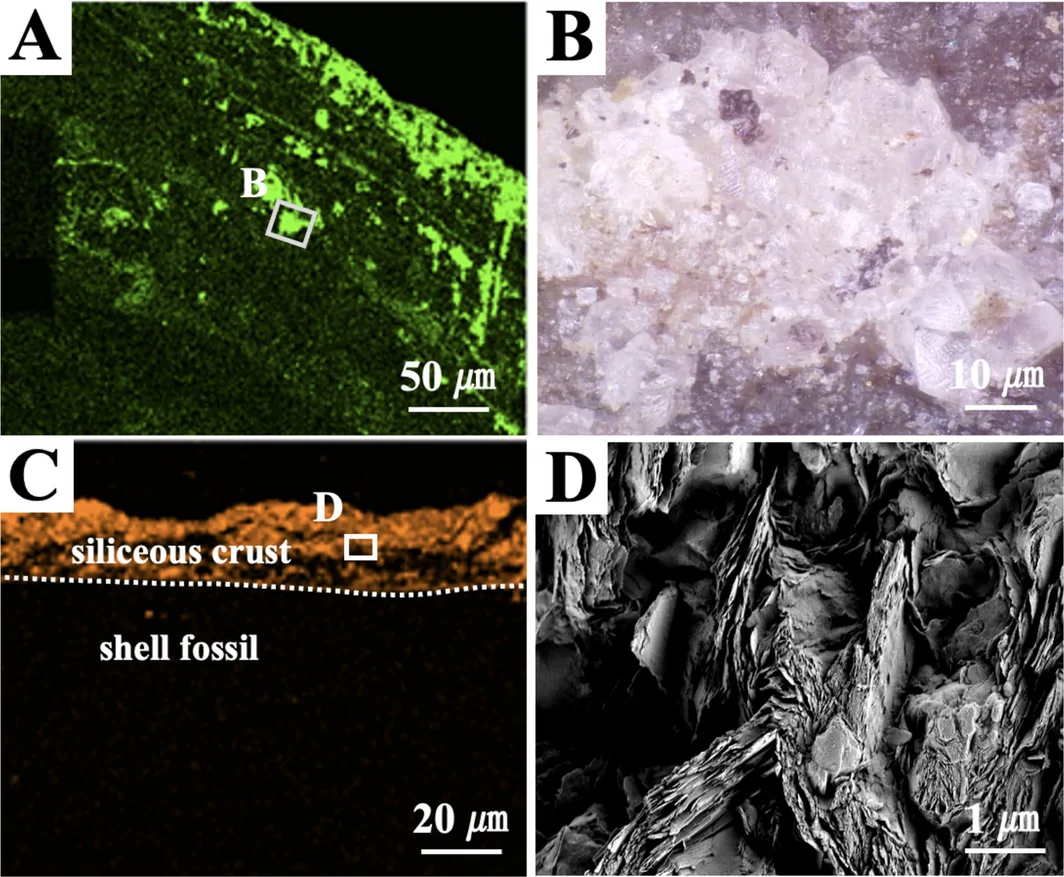
Spatial distribution of authigenic silica and microstructural boundaries at the shell–matrix interface. (A) Energy‐dispersive X‐ray spectroscopy (EDS) elemental mapping of silicon (Si, green) on the external fossil shell surface at the anteroventral margin (indicated by the arrow in Figure 1D), demonstrating a prominent, continuous siliceous coating over the outer shell architecture. (B) High‐magnification optical micrograph of the micro‐region demarcated by the white square B in A, revealing the translucent, microcrystalline character of the authigenic silica phase that encapsulates the outermost organic layers. (C) EDS elemental cross‐sectional mapping of silicon (Si, orange) spanning the boundary between the sedimentary host rock (matrix) and the fossilised shell. The white dashed line demarcates the sharp taphonomic interface between the protective siliceous crust and the underlying shell fossil. (D) High‐resolution FE‐SEM micrograph showing a fractured cross‐section along the maximum growth axis (corresponding to the section plane in Figure 1D) of the region demarcated by white square D in C. The image details the microcrystalline, sheet‐like authigenic silica aggregates that constitute the protective siliceous crust at the shell–matrix interface.

Furthermore, cross‐sectional EDS analysis spanning the shell‐to‐sediment boundary elucidated the precise spatial limits of this mineralisation. A distinct segregation of silicon was localized within the adjacent sedimentary matrix and the outermost boundary zone, establishing a sharp, highly resolved taphonomic interface (demarcated by the dashed line) between the protective siliceous crust and the underlying host shell fossil (Figure 4C). Within this protective siliceous boundary zone, high‐resolution FE‐SEM observations further revealed the microcrystalline, sheet‐like authigenic silica aggregates that form the protective crust (Figure 4D). The secondary mineral precipitates within this micro‐domain formed a rigid framework without causing structural disruption or overprinting of the primary molluscan microstructures.

## 4 Discussion

### 4.1 Molecular Integrity and the Longevity of Freshwater Chitin

The identification of chitinous remnants within the Cretaceous bivalves of the Kitadani Formation constitutes a significant expansion of the known molecular record (Briggs 2003; Briggs and Summons 2014). While chitin has been documented in various Palaeozoic and Mesozoic marine Lagerstätten, such as the Carboniferous Cyclus (Gupta et al. 2006) and Jurassic decapod fossils (Wysokowski et al. 2014), our findings represent the oldest definitive evidence of chitin preservation in a continental freshwater succession. The high degree of molecular fidelity—evidenced by the single Amide I band (1650 cm^−1^) diagnostic of the *β*‐polymorph, the *β*‐(1,4) C–O–C bridge (1157 cm^−1^), and the *β*‐glycosidic linkage at 896–898 cm^−1^ (Figure 3; Duarte et al. 2002; Kumirska et al. 2010), together with the specific fluorochrome response (Figure 2A,B; Muzzarelli 2013)—demonstrates that the *β*‐chitin scaffold has remained structurally intact despite over 120 million years of diagenesis (Cody et al. 2011).

It should be noted that the organic matrix in molluscan shells is not composed of pure chitin but rather exists as an insoluble composite framework—traditionally termed conchiolin or the “conchix” concept—wherein chitin microfibrils are intimately cross‐linked with structural proteins (Ehrlich, Janussen, et al. 2008; Ehrlich, Koutsoukos, et al. 2008; Marin et al. 2012). While our histochemical fluorochromes (CFW and CR) and FT‐IR amide absorption bands specifically verify the molecular preservation of the β‐linked chitin scaffold (Duarte et al. 2002; Muzzarelli 2013), the exceptionally preserved honeycomb‐like architecture in *N. soni* represents a remnant of this broader insoluble organic matrix. It is likely that recalcitrant proteinaceous components may have also contributed to the overall structural integrity and diagenetic resistance of the framework, which was subsequently stabilized by early‐stage silicification.

The absence of these molecular signatures in the surrounding sedimentary matrix confirms that the detected organics are indigenous to the shell and not the result of recent contamination or secondary infiltration. Such longevity challenges the traditional view that chitin is too labile to persist in freshwater environments (Gooday 1990), where microbial activity and fluctuating hydrochemistry typically accelerate the hydrolytic breakdown of nitrogenous organic matter (Flannery et al. 2001).

In the present study, the identification of indigenous chitin within the Cretaceous periostracal fossil is supported by multiple complementary lines of evidence, including high‐resolution ultrastructural congruence (Figure 2C–F), dual‐fluorochrome histochemical specificity validated against unstained autofluorescence controls (Figure 2A,B; Figure S1), and diagnostic FT‐IR absorption bands reflecting both the polysaccharide backbone (β‐glycosidic linkage at 896–898 cm^−1^) and associated amide modes (Figure 3). While destructive enzymatic digestion assays (e.g., using chitinases) and mass spectrometric characterisation (such as ESI‐MS) represent gold‐standard benchmarks for isolating and sequencing pure chitin chains in modern and exceptionally preserved subfossil materials (e.g., Bailey et al. 2025), the intimate micro‐scale encapsulation and inorganic cross‐linking within our rigid silica–chitin hybrid framework (Figure 4) present intrinsic steric hindrance against macromolecular enzymatic penetration without aggressive chemical desilicification. Further investigations employing micro‐scale extraction coupled with advanced mass spectrometry will be valuable to elucidate the fine‐scale fragmentation and cross‐linking pathways of Mesozoic biopolymers.

### 4.2 The ‘Taphonomic Shield’: Early‐Stage Silicification as a Preservative Mechanism

Multifaceted studies have recently highlighted the exceptional preservation of molluscan fossils yielded from the Kitadani Formation (Asato et al. 2022; Yoshimura, Nakayama, et al. 2026; Yoshimura, Nakayama, Asato, et al. 2026). We propose that the silica coating functioned as a ‘taphonomic shield’ (Butterfield 2003), providing a dual‐layered protection against degradative processes (Figure 5). Key morphological evidence for this mechanism is the preservation of periostracal honeycomb‐like templates (ca. 30 μm in diameter; Yoshimura, Nakayama, et al. 2026). These polygonal frameworks, morphologically congruent with those of the extant *Nodularia douglasiae* , indicate that silicification was rapid enough to ‘mummify’ the delicate organic architecture before it could undergo microbial decay or structural collapse.

**FIGURE 5:**
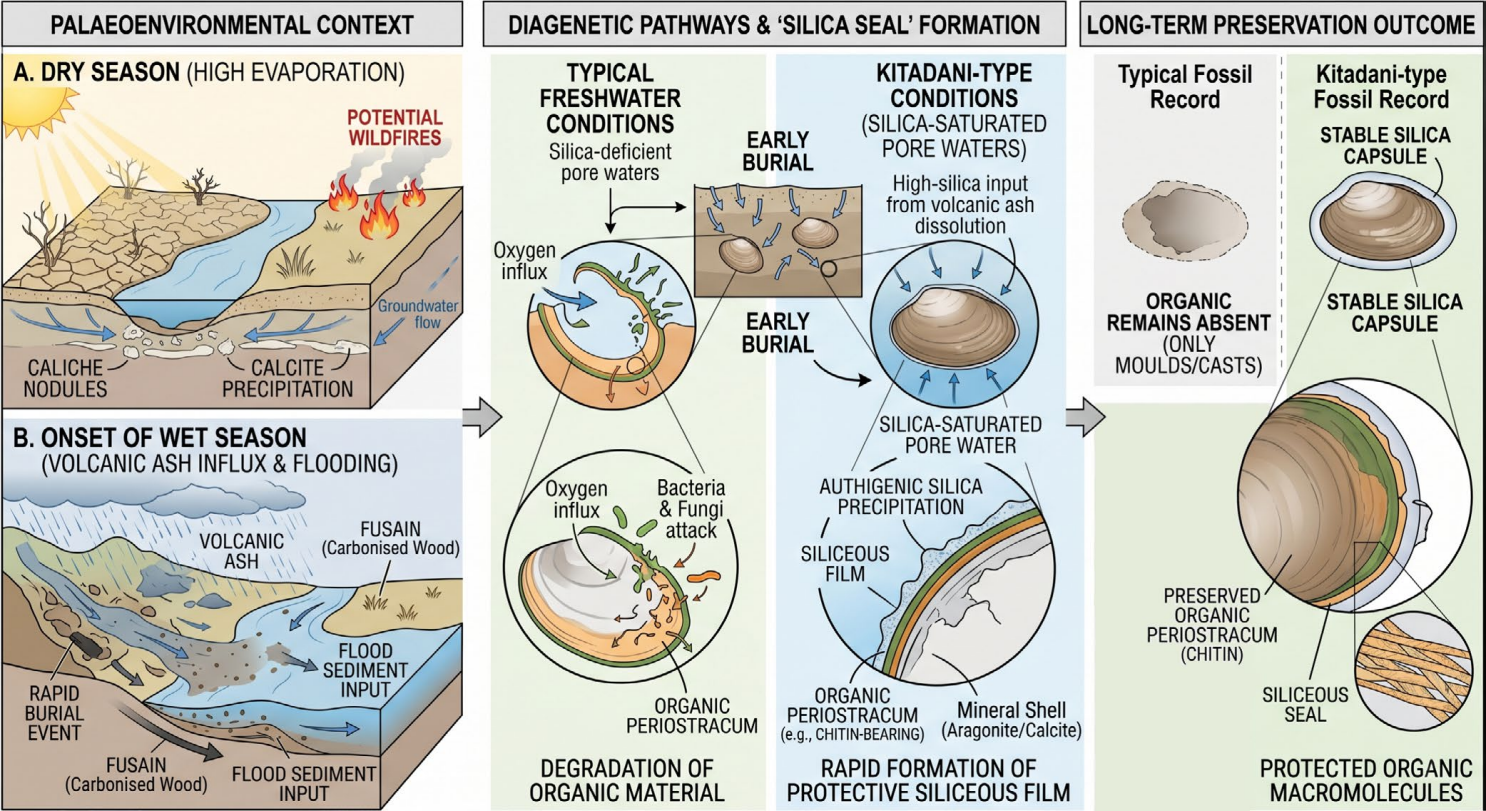
Conceptual taphonomic model for the molecular preservation of organic macromolecules in the Kitadani Formation. (A, B) Palaeoenvironmental context: (A) Dry season characterised by high evaporation, groundwater fluctuation, and localized calcite precipitation (caliche nodules). (B) Onset of the wet season inducing high‐energy flooding, volcanic ash influx, and rapid burial of organic matter alongside carbonised wood (fusain). Middle panel. Diagenetic pathways and ‘silica seal’ formation: Divergent taphonomic trajectories following early burial. Under typical silica‐deficient freshwater conditions (left), oxygen influx facilitates microbial degradation of the organic periostracum by bacteria and fungi. Under silica‐saturated Kitadani‐type conditions (right), rapid dissolution of volcanic ash yields silica‐rich pore waters, driving authigenic silica precipitation as a protective micro‐scale film over the chitin‐bearing periostracum. Right panel. Long‐term preservation outcome: Comparison of fossilisation products. Typical freshwater diagenesis results in total organic loss, leaving only inorganic sedimentary moulds and casts. In contrast, the Kitadani‐type record forms a stable silica capsule that physically and chemically shields the original chitinous microfibrils from late‐stage diagenetic leaching.

Firstly, the early‐stage precipitation of silica likely occurred prior to extensive microbial colonisation of the shell matrix (Toporski et al. 2002). By sealing the shell, this mineralisation created a physical barrier that prevented chitinolytic enzymes—typically 40–100 kDa in size (Merzendorfer and Zimoch 2003)—from accessing their substrate. Furthermore, as noted in taphonomic models of shell silicification (Butts 2014), the rapid formation of a siliceous crust on the shell can effectively restrict fluid exchange, thereby precluding pervasive silicification of the shell interior while isolating the internal organic matrix. This steric hindrance is crucial, as enzymatic degradation is often the primary driver of organic loss in the early post‐mortem interval (Briggs and McMahon 2016). The feasibility of such rapid, micro‐scale silica encapsulation is also supported by experimental silicification studies of plant tissues (Ballhaus et al. 2012; Läbe et al. 2012; Hellawell et al. 2015), which demonstrate that dissolved silica can quickly template onto organic substrates before extensive structural decay occurs.

### 4.3 Chemical Stabilisation and the Silica–Chitin Complex

The chemical environment at the silica‐organic interface played a vital stabilising role (Leo and Barghoorn 1976). Our decalcification experiments revealed that the resulting insoluble organic framework (IOF) was a self‐supporting, rigid silica–chitin hybrid rather than a soft residue. This suggests that dissolved silica was adsorbed onto organic surfaces through hydrogen bonding and specific cation bridging (Butts and Briggs 2010; Butts 2014), effectively impregnating the microfibrils at a micrometre scale.

Natural structural composites of silica and chitin are well‐documented in biomineralisation, particularly among diatoms and poriferans (Ehrlich, Janussen, et al. 2008; Ehrlich, Koutsoukos, et al. 2008; Durkin et al. 2009). In diatoms, chitin microfibrils form structural components associated with the biosiliceous frustule, acting as organic scaffolds during cell wall assembly and morphogenesis (Durkin et al. 2009). Similarly, biosilica–chitin frameworks in sponges provide structural support and facilitate controlled silica deposition (Ehrlich, Janussen, et al. 2008; Ehrlich, Koutsoukos, et al. 2008; Ehrlich et al. 2013). At the molecular interface, the hydroxyl and amide functional groups of the chitin polymer readily interact with silanol groups (≡ Si–OH) via hydrogen bonding and electrostatic interactions, providing a stereochemically favourable template for silica condensation. Although the silica–chitin framework observed in *N. soni* originated through secondary diagenetic mineralisation rather than primary biosilicification, these biological analogues, together with our recovery of a rigid silica–chitin hybrid framework following decalcification (Figure 4), support the hypothesis that dissolved silica can effectively template onto organic chitin networks via structural and chemical compatibility.

In the specific hydrochemical regime of the Cretaceous Kitadani fluvial system, which was likely enriched in dissolved silica due to contemporaneous volcaniclastic input (Sano 2015), this rapid mineralisation effectively encapsulated the chitinous architecture (Schopf et al. 2002). Although the protective coating is currently present as microcrystalline silica aggregates, this phase likely originated from an initial, non‐crystalline or amorphous silica precursor (e.g., opal‐A/opal‐CT) that rapidly templated onto the organic periostracal substrate before undergoing diagenetic recrystallisation. This process is analogous to the preservation of soft tissues in marine pyritisation (Briggs and McMahon 2016), but notably occurs here via silicification, highlighting a distinct and robust pathway for molecular fossilisation in continental basins.

### 4.4 Environmental Drivers and Broader Implications

The discovery of silica‐protected chitin in a freshwater setting suggests that our current understanding of the ‘molecular fossil record’ may be influenced by a significant taphonomic bias (Allison and Bottjer 2010). Marine environments have historically been the focus of molecular palaeontology (Peterson et al. 2007), yet the Kitadani Formation demonstrates that specific authigenic mineralisation events can override the deleterious effects of freshwater diagenesis (Kubota 2005; Yabumoto 2005).

The co‐occurrence of caliche nodules and carbonised wood (fusain) within the lithostratigraphic column (Figure 1E) provides key evidence for the dynamic palaeoclimatic regime that drove this molecular preservation (Figure 5A,B). The presence of pedogenic caliche nodules signifies a highly seasonal climate marked by a pronounced dry season with elevated evaporation rates (Figure 5A). During these prolonged arid intervals, parched terrestrial vegetation became highly susceptible to ignition, triggering widespread wildfires that generated the macroscopic fusain observed in the sequence. We propose that the subsequent onset of the wet season induced catastrophic, high‐energy fluvial flooding (Figure 5B). These initial flash floods rapidly flushed both the silica‐rich, contemporaneous volcanic ash and massive volumes of sediment into the basin. This specific environmental trigger not only facilitated the instantaneous, rapid burial of the bivalves but also introduced highly silica‐saturated pore waters, effectively initiating the authigenic precipitation of the protective ‘silica seal’ before extensive microbial overprinting could occur (Figure 5).

If such volcaniclastic‐infused, seasonal depositional systems are more prevalent in terrestrial basins than previously recognised (e.g., Lockley and Rice 1990; Sano 2015), it is probable that other organic‐walled fossils remain to be discovered within similar ‘silica‐sealed’ contexts (e.g., Strang et al. 2016). Our results advocate for a systematic re‐evaluation of freshwater invertebrate remains, using high‐resolution analytical techniques such as FE‐SEM‐EDS, CLSM, and TEM (Ehrlich, Janussen, et al. 2008; Ehrlich, Koutsoukos, et al. 2008), to identify recalcitrant biopolymers in the continental record.

## 5 Conclusions

Our multi‐analytical investigation provides definitive evidence that chitinous biopolymers can persist for over 120 million years within dynamic continental fluvial environments. The discovery of preserved honeycomb‐like periostracal templates in *Nakamuranaia soni* provides a crucial structural‐molecular link, representing the oldest record of indigenous chitin within a strictly freshwater fluvial Lophotrochozoan. The exceptional fidelity of the amide I and II bands, coupled with the absence of such signatures in the sedimentary host rock, confirms the indigenous nature of these organics and challenges the long‐standing assumption that chitin is too labile to survive the high microbial turnover of freshwater diagenesis. We conclude that early‐stage silicification acted as a ‘taphonomic shield’, creating a rigid, self‐supporting silica–chitin hybrid that provided both steric hindrance against enzymatic degradation and chemical stabilisation against leaching. These findings indicate that continental fluvial successions, particularly those influenced by contemporaneous volcanism, possess an untapped potential for molecular palaeontology. By integrating high‐resolution spectroscopy with comparative morphology, this study establishes a new framework for identifying hidden molecular treasures in the terrestrial fossil record, suggesting that the molecular window into extinct life is broader than previously recognised.

## Funding

This work was supported by JSPS KAKENHI Grant Number 23KJ0818, 25K24907, and 26K02079.

## Conflicts of Interest

The authors declare no conflicts of interest.

## Supporting information


**Figure S1:** Epifluorescence micrograph of the unstained fossilised periostracum of *Nakamuranaia soni* (negative control). High‐magnification epifluorescence image acquired under ultraviolet excitation (excitation: 365 nm, emission: 447 nm) without histochemical staining. The image was captured using acquisition parameters identical to those used for the Calcofluor White (CFW)‐stained sample in Figure 2A (exposure time: 1/500 s). The unstained organic matrix exhibits only faint, negligible background autofluorescence, confirming that the intense blue fluorescence resolved in Figure 2A is specific to CFW binding rather than non‐specific proteinaceous or mineral autofluorescence.

## Data Availability

The dataset supporting this research is openly available in the Dryad Digital Repository at https://doi.org/10.5061/dryad.gqnk98t10. For the purpose of peer review, the dataset supporting this manuscript is available at the following secure URL: http://datadryad.org/share/R1hMw3_FZEm_tQm‐NNJC8q9An2HAf3qdr1Jj4xtrBX8.
